# Exploring the relationship between cyberloafing and innovativeness among nurses in research hospitals: a cross-sectional study in Turkey

**DOI:** 10.1186/s12912-024-02008-6

**Published:** 2024-06-03

**Authors:** Semra Aciksoz, Merdiye Sendir, Nurdan Yalcin Atar, Nesibe Simsekoglu, Kursad Nuri Baydili

**Affiliations:** 1 Hamidiye Faculty of Nursing, University of Health Sciences, Selimiye District, Tibbiye Street No: 38, 34668 Uskudar, Istanbul, Turkey; 2 University of Health Sciences, Hamidiye, Vocational School of Health Services, Selimiye District, Tibbiye Street No: 38, 34668 Uskudar, Istanbul, Turkey

**Keywords:** Cyberloafing, Innovativeness, Nurse

## Abstract

**Background:**

Although cyberloafing, which refers to employees' use of the internet for private purposes outside of work, is seen as a negative behavior; Positive effects on areas such as individual development, learning opportunity, job satisfaction, productivity, change, organizational output, and innovation have also been reported. This study aims to investigate whether there is a significant relationship between cyberloafing and the innovativeness levels of nurses working in research hospitals in Turkey.

**Methods:**

This analytical study consisted of 230 nurses. Data were collected using a nurse information form, Cyberloafing Scale, and Individual Innovativeness Scale. Data analysis was carried out using descriptive, comparative, and correlational statistics.

**Results:**

Nurses had low levels of minor and severe cyberloafing scores and skeptical innovativeness. There was no correlation between cyberloafing and individual innovativeness.

**Conclusions:**

More conscious cyberloafing should be encouraged through institutional arrangements, which can improve nurses' individual and innovative professional aspects.

## Background

One of the areas most affected by the changes in science and technology is health treatment and care practices. In this area, it is expected that innovative services, products, or processes will be developed for the benefit of society and patients. Innovative behavior style refers to the ability of individuals to define their problems with an innovative perspective and to create individual solutions [1, 2].

Nurses are in an important position to produce new and innovative solutions to problems, as they are in direct contact with patients and are at the forefront of the healthcare system. Nurses engage in creative activities focused on improving care outcomes, reducing the health system's cost, and increasing satisfaction. Innovative nursing practices can be developed in all areas, especially in areas such as care practices, research, education, technology and public health. These innovative activities and health systems play an important role in improving the health level of individuals and communities [3–5].

Innovation in Nursing; It is a process in which new approaches to patient care are developed and translated into viable outcomes [3, 4, 6]. The International Council of Nurses defines the theme of 2009 as "nursing and innovation" and argues that nurses should be pioneers in innovative healthcare practices to provide quality healthcare to society. Nurses, who focus on the concept of humans, should be open to innovations brought by technology, based on science, and renew themselves according to economic, social, and social developments. For nurses to meet their care needs and fulfill their modern nursing roles with an innovative approach, they are expected to have an innovative mindset, not to oppose this approach, and to consider and implement innovations. In addition, nurses; need to be well-equipped, and have a leadership role and risk-taking personality traits. While the support of administrators is of great importance in the development of the innovation culture of nurses, the increase in workload is one of the factors that negatively affect their innovative attitudes. With its innovative feature, nurses have the opportunity to develop both individually and professionally [4, 7–11]. In the literature, there is the view that nurses are successful in being innovative and productive. In a study conducted by Baumann (2011), it was determined that nurses are more prone to innovation [4, 11, 12].

The use of communication and information technologies can play an important role in individuals' following innovations in their fields, accessing new information, and learning. Studies have also shown that there is a significant relationship between individuals' innovativeness levels and their use of information technologies [7, 13–15]. Nurses frequently use tools such as internet connection, telephone, and computer to record patient data and care practices, access patient information, and access and benefit from scientific literature and mobile applications. During working hours of all corporate and personal vehicles providing internet access; Using it for non-work-related purposes such as watching videos, communicating, surfing social networking sites, or playing online games is defined as cyberloafing by Robinson and Bennett (1995) [16–18].

Anandarajan et al. (2004) discussed the cyberloafing behaviors of employees in four groups. These; Prohibited practices include activities that do not benefit the organization but aim to obtain business-related information, have fun, and may pose risks within the scope of uncertain activities [1, 19, 20].

Although the uncertainty of the exact factors affecting cyberloafing behavior remains valid, there are studies indicating that cyberloafing behavior is common regardless of profession, working hours, or even age and gender [21]. Jiang et al. (2020) developed a theoretical model to understand under which conditions the use of personal technology in the workplace can increase or decrease the job performance of the employee. In this model, among the factors affecting the job performance of the employees, especially the 'manager attention' is emphasized [22].

According to 2022 internet usage data in Turkey, the regular internet usage rate is 82.7%. 78.6% of women and 86.9% of men use the internet regularly. The rate of using the Internet in the workplace is 94.9%. Cyberloafing activities become uncontrollable unless the usage policy of the internet, whose usage is increasing day by day, is determined. In this context, there are institutions where access to the internet is provided or blocked with approval to ensure the safety of internet use [23, 24]. As a matter of fact, in the study of Lim et al. (2012), it is argued that when employees have a strong locus of control, cyberloafing behaviors will greatly decrease and productivity will increase [25].

In the studies, it has been emphasized that cyberloafing may cause economic losses by reducing productivity due to ineffective use of time and resources in the workplace, alienation from work, procrastination, decreased productivity, decreased organizational/individual performance, technology addiction and patient safety [1, 26–29]. Furthermore, the detrimental effects of cyberloafing on organizational trust, organizational justice and work engagement have been empirically validated [5]. Anesthesiologists of the American Nurses Association (ANAA; 2013) also published a report stating that cyberloafing behavior will create safety problems for patients. Some governments have made decisions to prevent civil servants from accessing the Internet on corporate computers because employees engage in cyberloafing behavior for an average of two hours a day and the annual loss of more than 759 billion dollars as a result of cyber information sharing [18, 21, 30]. 

The attention and sensitivity of health professionals in the provision of health services directly affect human health. Distractions that may arise when nurses use personal communication devices during their care practices and may lead to substandard nursing practices are met with concern all over the world. In the Emergency Care Research Institute's technology hazard report, mobile device use during maintenance practices is described as a distracting hazard. There are also studies showing that nurses' cyberloafing behaviors harm healthcare practices and patient safety [7, 21, 28]. In Patterson's (2012) study, it was reported that operating room managers complained about the distraction of employees who engage in cyberloafing behavior [31].

Although the negative consequences of cyberloafing are emphasized; positive effects on stress, motivation, job satisfaction, employee satisfaction, innovation, and learning opportunities are also stated [1, 7, 18, 21]. Cyberloafing can be effective in facilitating nurses' access to innovative practices, medical references, clinical tools, and patient information, increasing their performance and reducing their stress. In particular, determining the effect of cyberloafing behavior on innovation is important in terms of nursing science, which aims to provide quality healthcare services. Finding limited and contradictory results regarding the effects of nurses' cyberloafing behaviors on innovativeness in the literature reveals that this issue should be investigated in more depth [1]. In Elhanafi's study, it was determined that there was a negative relationship between nurses' cyberloafing and their "trust, justice, and commitment" status. It was stated that this situation negatively affected the innovative behaviors and quality of care of nurses [32]. Palladan (2018), in a study examining the effect of lecturers' cyberloafing behaviors on innovativeness, determined that lecturers' cyberloafing activity between lectures had negative effects on innovation and job performance [33]. Although there are generally negative results in studies examining the effect of cyberloafing behavior on innovation, these results allow individuals to increase their awareness of cyberloafing behaviors. In the study of Chen et al. (2022), in which they examined cyberloafing behavior based on the dual system theory, it was stated that the increase in participants' awareness of cyberloafing decreased their cyberloafing times [34]. The results obtained from this research will also enable individuals to increase their awareness of cyberloafing levels and allow managers to take measures in this direction.

According to Van Doorn (2011) engaging in cyberloafing behavior; has four purposes: habit, renewal, abuse, and personal development behaviors [1, 10, 27]. It has gained importance to examine the purpose for which nurses, whose main goal is to provide quality health care services, perform cyberloafing behavior, the levels of cyberloafing, and the factors affecting them [1, 26].

This study is important because the fact that nurses' cyberloafing behaviors are directly related to low-level healthcare services will pose a risk to patient safety. It is thought that the findings obtained from this study will guide the development of policies and guidelines regarding personal communication device use.

Considering that cyberloafing behavior, which is generally perceived as a threat, can also provide an opportunity to follow innovations and develop, it is important to develop suggestions for institutions, managers, and personnel. In this context, international organizations also state that there is a need for various regulations for nurses to use the Internet consciously. Considering the limitations of studies on cyberloafing and innovativeness levels in nurses, it is thought that this study will fill the gap in the field by bringing a new perspective. The research aims to investigate whether there is a significant relationship between cyberloafing and innovativeness levels of nurses working in research hospitals in Turkey. The main research questions were as follows:

- What are the cyberloafing and innovativeness levels of nurses?
- Is there a relationship between cyberloafing behaviors and individual innovativeness characteristics among nurses?

## Methods

### Study design and location

The study utilized a descriptive, cross-sectional, correlational design. The research was conducted in three research hospitals in Turkey. These hospitals were chosen because they provide competent, safe, and quality health care services, have a large number of patient applications, and computers are used in each clinic. Hospital A is a Gynecology and Pediatrics Hospital. It has a total capacity of 285 nurses and 352 beds. The total number of nurses in hospital B, which is a general research hospital, is 355 and its bed capacity is 709. Finally, Hospital C has a total capacity of 310 nurses and 698 beds.

### Setting and samples

The population of the study consisted of 950 nurses employed in three teaching and research hospitals in Istanbul, Turkey. The sample consists of nurses working in three different hospitals and agreeing to participate in the research. In the research, the number of observations that should be included in the sample was calculated using the data of the study conducted by Palladan (2018) [35]. As a result of calculations; It was determined that maximum power would be reached by including 196 individuals in the sample (R0 = 0; R1 = 0.639; α = 0.05). Data collection was completed with 230 nurses who selected by simple random sampling and agreed to participate in the research. Following the completion of the study, a post hoc power analysis was conducted to assess the adequacy of the sample size achieved. The power analysis indicated that the total sample size was sufficient, with an effect size of 1.09, 99.8% power, and a margin of error of 0.05.

### Inclusion and exclusion criteria

The study recruited participants using a convenience sampling design with inclusion–exclusion criteria.

Inclusion criteria:

- Working as a nurse at the relevant hospitals at the time of the research.
- Providing direct patient care,
- Must have given consent to participate in the study.
- Using a smartphone.

Exclusion criteria:

- Withdrawal of participants from the study.

### Measures

The data were collected using a nurse information form, the Individual Innovativeness Scale (IIS), and the Cyberloafing Scale (CLS).

### Nurse information form

The form was developed by the investigators after reviewing the relevant literature [4, 13, 26, 27]. It consists of 21 questions related to the nurses’ socio-demographic characteristics and internet usage characteristics.

### Individual innovativeness scale

This scale was developed by Hurt et al. in 1977 to determine the level of innovativeness that individuals would generally have, along with the category of innovativeness to which they belong. The Turkish adaptation of the scale was conducted by Sarioglu and Altuntas (2014), is a Likert-type scale consisting of 18 items and 3 subdimensions (opinion leadership, resistance to change, and risk-taking) [36]. Five different categories are determined by the chase characteristics of individuals. Participants achieving scores of over 82 are determined to be “innovators”, those with scores between 75 and 82 are “pioneers”, those scoring 66–74 are “inquirers”, those obtaining scores between 58 and 65 are called skeptics”, and those who obtain 57 and below are stated to be “traditionalists”. The Cronbach’s alpha value of the original scale was found 0.82 [16]. Cronbach alpha value of our study was also found to be 0.81.

### Cyberloafing scale

The CLS was developed by Blanchard and Henle (2008) to measure the frequency of individuals' cyberloafing behaviors. It was adapted to Turkish by Opucu and Yildiz (2014) [37]. It has 14 items in two subdimensions. These subdimensions were determined as minor cyberloafing behaviors (checking, receiving, and sending non-work-related emails, etc.), and serious cyberloafing activities (downloading music or films, joining chat rooms, etc.). The Cronbach’s alpha value of the original scale was found 0.89 [37]The responses to the items in the scale are of the five-point Likert type, with scores ranging from 1 for “never” to 5 for “very frequently”. An increase in statement values from1 to 5 indicates an increase in the frequency of cyberloafing behaviors.

### Data collection procedure

The study was evaluated by the ethics committee of Zeynep Kamil Research Hospital and found appropriate in terms of medical ethics. A pilot test was done to check the clarity and simplicity of instrument items. No ambiguity was reported.

The purpose of the study was explained by the researcher by interviewing the director of each hospital. Then, the nurses in each department informed the managers about the purpose of the study. After screening nurses according to inclusion–exclusion criteria, a list of nurses was obtained from nurse managers to identify all potential participants. Nurses who met the eligibility criteria of the study applied for recruitment. The purpose of the study was explained to the nurses participating in the study. Participants were informed that participation in the study was entirely voluntary and that they could withdraw from the study at any time. The benefits, risks and expected roles of participating in the research were explained.

The privacy of the participants was always respected. Only the researcher has access to all copies of the participant information forms and completed questionnaires. Likewise, the anonymity of the participants was preserved. No personally identifiable information was collected from the participants. Participants did not undergo any experimentation or intervention.

Forms were given to each nurse after an explanation about the purpose, contents, scope, and duration of the study, as well as what was expected from the study. Research data were collected in one time and in approximately 6–7 min by face-to-face interview method between October and December 2018.

### Data analysis

The data were analyzed using SPSS for Windows software, version 25.0 (Statistical Package for the Social Science for Windows). Numbers, percentages, arithmetic means, and standard deviations were used for descriptive data. The normality of variable distributions was measured with the Kolmogorov–Smirnov test and was found to be normal. For comparison of the demographic variables with the scale scores, independent samples t-test and ANOVA test was conducted. For comparisons between two continuous variables, Pearson correlation was used. Factors affecting individual innovativeness were determined by linear regression. In the study significance was set at *p* < 0.05.

## Results

The participants in this study were found to be between the ages of 19 and 58, with 46.5% belonging to the 25–29 age group. It was established that 88.3% of the participants were female, 66.1% had obtained undergraduate qualifications, 59.2% were ward nurses, and 66.5% had been employed for a period of 0–5 years. It was determined that 51.3% of the nurses were satisfied with their career, and 95.2% of them were engaged in the implementation of innovations related to the nursing profession (Table 1).

**Table 1:** Socio-demographic and professional characteristics of nurses (*n* = 230)

Age	n	%
19–24	53	23.0
25–29	107	46.5
30–35	33	14.3
36–40	19	8.3
40 >	18	7.8
**Gender**		
Female	203	88.3
Male	27	11.7
**Education**		
Vocational School	41	17.8
Associate degree	6	2.6
Graduate	152	66.1
Postgraduate	31	13.5
**Working year**		
5 years <	153	66.5
6–10 years	37	16.1
11 years >	40	17.4
**Workplace**		
Intensive care	43	18.7
Surgical clinic	57	24.8
Internal clinic	67	29.1
Emergency	19	8.3
Other	44	19.1
**Working Shift**		
Day Shift	56	24.3
Day Shift + Night Shift	167	72.6
Night Shift	7	3.0
**Weekly Working Time**		
40 h <	65	28.3
41 h >	165	71.7

The internet usage characteristics of the nurses were as follows: 35.6% used the internet for 1–2 h per day, 64.8% used it for less than one hour during working hours, 68.3% used it for communication, 60.4% of them used it for research and reading news, and 92.2% used the mobile phones. Of nurses, 90.4% stated that going on the internet during working hours did not affect their work plans or practice (Table 2).

**Table 2:** Internet usage characteristics of nurses (n = 230)

Internet Usage Time (per day)	n	%
No	1	0.4
< 1 h	29	12.6
1–2 h	82	35.6
2–3 h	64	27.8
> 3 h	54	23.4
**Internet Usage Time Range**		
06–12	46	20.0
12–18	70	30.4
18–24	42	18.3
24–06	72	31.3
**Internet Usage Time during Working Hours**		
No	16	7.0
< 1 h	149	64.8
1–2 h	43	18.7
2–3 h	10	4.3
> 3 h	12	5.2
**Purpose of Internet Usage during Working Hours**^a^		
Communication	157	68.3
Research /	139	60.4
Games/Entertainment	32	13.9
Shopping	30	13.0
**Internet Device Used during Working Hours**^a^		
Mobile phones	212	92.2
Computer in workplace	86	37.4
Personal computer	8	3.4
**Influence of Internet Usage during Working Hours on Work Plans**		
Yes	9	3.9
No	208	90.4
Partially	13	5.6

^a^Multiple options are selected

The nurses’ CLS scores were 2.63 ± 1.02 for the serious cyberloafing subdimension, 2.61 ± 0.93 for the minor cyberloafing subdimension, and 2.62 ± 0.90 for the total CLS mean score. These results show that the nurses sometimes displayed cyberloafing behavior. The nurses’ mean IIS score was 60.34 ± 7.07. The nurses’ innovativeness characteristics were among the “skeptics” skeptics (Table 3).

**Table 3:** Scale scores and internal consistency

	**α**^b^	**Min–Max**	**X ± SS**^a^
**CLS**			
Important cyberloafing	0.720	1.00–4.91	2.63 ± 1.02
Unimportant cyberloafing	0.890	1.00–4.90	2.61 ± 0.93
Total	0.878	1.00–4.91	2.62 ± 0.90
**IIS**	0.747	43.00–90.00	60.34 ± 7.07

*CLS* Cyberloafing Scale, *IIS* Individual Innovativeness Scale

^a^Mean ± Standard deviation

^b^α = Cronbach Alpha

CLS mean scores of nurses who were aged 36 and over were statistically significantly lower and they rarely displayed cyberloafing behavior (*p* = 0.007). IIS mean scores of male nurses were statistically significantly higher (*p* = 0.008). Nurses with 11 or more years of work experience had lower CLS mean scores and rarely displayed cyberloafing behavior (*p* = 0.001). Nurses’ who were very satisfied with their profession had higher IIS mean scores and they were the in “inquirers” category (*p* = 0.001). Nurses’ who stated being indecisive following the innovations had statistically significantly lower IIS mean scores (*p* = 0.002). Nurses’ who used the internet for more than three hours a day had higher CLS mean scores and sometimes displayed cyberloafing behavior (*p* = 0.002) (Table 4).

**Table 4:** Comparison of the CLS and IIS total score according to nurses' socio-demographic and internet usage characteristics (*n* = 230)

	**n**	**%**	**CLS** X ± SS*	**IIS** **X ± SS**^a^
**Age (years)**				
19–24	53	23.0	2.58 ± 0.80	60.81 ± 7.62
25–29	107	46.5	2.80 ± 0.92	59.80 ± 6.18
30–35	33	14.3	2.52 ± 0.95	59.90 ± 5.39
36–40	19	8.3	2.07 ± 0.70	59.42 ± 8.30
40 >	18	7.8	2.32 ± 0.88	63.88 ± 10.61
^b^F/p			3.661;0.007*	1.468;0.213
**Gender**				
Female	203	88.3	2.61 ± 0.90	59.88 ± 6.80
Male	27	11.7	2.63 ± 0.91	63.74 ± 8.18
^c^t/p			-0.113;0.910	-2.696;0.008*
**Education**				
Vocational school	41	17.8	2.30 ± 0.70	60.90 ± 6.93
Associate degree	6	2.6	2.98 ± 1.26	57.00 ± 4.51
Graduate	152	66.1	2.70 ± 0.89	60.17 ± 7.37
Postgraduate	31	13.5	2.51 ± 1.00	61.06 ± 6.12
^b^F/p			2.636;0.510	0.667;0.573
**Workplace**				
Intensive care	43	18.7	2.27 ± 0.68	61.19 ± 5.33
Surgical Clinic	57	24.8	2.60 ± 0.92	60.52 ± 8.13
Internal Clinic	67	29.1	2.87 ± 0.84	59.09 ± 5.06
Emergency	19	8.3	2.72 ± 1.08	58.66 ± 3.86
Others	44	19.1	2.29 ± 0.74	63.00 ± 5.58
^b^F/p			2.191;0.071	1.149;0.334
**Working Type**				
Day	56	24.3	2.44 ± 0.87	61.03 ± 6.72
Day + Night	167	72.6	2.67 ± 0.90	60.13 ± 7.21
Night	7	3.0	2.70 ± 0.96	59.57 ± 6.92
^b^F/p			1.477;0.230	0.378;0.685
**Working year**				
5 years <	153	66.5	2.76 ± 0.90	60.36 ± 7.11
6–10 years	37	16.1	2.44 ± 0.91	60.34 ± 7.09
11 years >	40	17.4	2.20 ± 0.71	57.00 ± .0.01
^b^F/p			7.249;0.001*	0.955;0.387
**Job Satisfaction**				
I am not happy at all	17	7.3	2.76 ± 1.18	61.35 ± 8.68
I am not satisfied	23	10.0	2.53 ± 0.76	60.39 ± 6.45
Undecided	56	24.3	2.56 ± 0.93	59.69 ± 4.74
Satisfied	118	51.3	2.71 ± 0.85	59.00 ± 5.96
I am very pleased	16	6.9	2.06 ± 0.82	71.31 ± 10.79
^b^F/p			2.044;0.089	13.212; < 0.001*
**Following Innovations**				
Yes	129	56	2.62 ± 0.89	61.69 ± 7.80
No	13	5.5	2.73 ± 1.17	61.00 ± 8.13
Undecided	88	38.5	2.58 ± 0.87	58.25 ± 5.05
^b^F/p			0.162;0.851	6.581;0.002*
**Internet Usage Time (per day)**				
< 1 h	165	71.8	2.31 ± 0.86	61.90 ± 7.56
1–2 h	43	18.7	2.53 ± 0.94	59.06 ± 5.42
2–3 h	10	4.3	2.54 ± 0.75	61.60 ± 8.61
> 3 h	12	5.2	3.00 ± 0.91	59.90 ± 6.74
^b^F/p			5.087;0.002*	2.167;0.093

*CLS* Cyberloafing Scale, *IIS* Individual Innovativeness Scale

^a^Mean ± Standard Deviation

^b^F = ANOVA

^c^t = Independent Simple t test

As a result of the comparison of the total CLS and IIS mean scores, no statistically significant difference was found between total CLS (2.62 ± 0.90) and IIS (60.34 ± 7.07) mean scores (*r* = -0.031; *p* = 0.644).

As a result of multiple regression carried out to determine the factors affecting individual innovativeness in men and women; It has been determined that following innovations in women positively affects individual innovativeness (*p* = 0.002) (Table 5). In men, it was found that job satisfaction (*p* = 0.006) and following innovations (*p* = 0.042) positively affected individual innovativeness, but CLS negatively affected individual innovativeness (*p* < 0.001).

**Table 5:** Examining the factors affecting individual innovativeness

	B	SE	Beta	t	*p*	95% CI
(Constant)	3,077	0,144		21,349	< 0,001	2,793–3,361
Job Satisfaction	0,015	0,027	0,04	0,569	0,570	-0,038–0,069
Following Innovations	0,102	0,045	0,156	2,238	0,026*	0,012–0,191
CLS	-0,012	0,029	-0,028	-0,434	0,665	-0,069–0,044

^*^p < 0,05

## Discussion

This study was conducted to evaluate the relationship between nurses’ cyberloafing behaviors and individual innovativeness characteristics, with consideration for the influence of their characteristics.

The nurses’ mean total IIS score was 60.34 ± 7.07. Different from the previous studies, the mean total score obtained by the students on the ISS indicated that the nurses’ innovativeness characteristics were in the “sceptics” and they were low-level innovators [36, 38, 39]. The characteristics of individuals in this category are skepticism and distrust towards innovation. Sonmez and Yildirim (2014) revealed that nurses were not disposed to take risks, but they tended to implement current innovations [39]. Besides the individual and institutional characteristics, the nurses’ critical thinking for giving no harm to people and the desire to implement innovations after their results have become widespread may have been a factor in putting innovations into practice.

The CLS score total score was 2.62 ± 0.90 and subscale totals were 2.63 ± 1.02 for serious cyberloafing; 2.61 ± 0.93 for minor cyberloafing. The mean CLS scores showed that the nurses “sometimes” displayed both serious and minor cyberloafing behaviors. Similarly, several previous studies found that cyberloafing behaviors were at low levels among nurses [13, 27, 36, 40–42]. These findings can be explained by the fact that nurses have heavy workloads. In the literature, it is stated that the level of cyberloafing is low both in nurses [43] and in other healthcare professionals [9, 44]. However, in some studies, the level of cyberloafing was found to be high [1, 45] or moderate [1, 45]. There are also studies reporting that the cyberloafing levels of health sector workers are lower than those of textile sector workers [1].

Significant differences were found between the nurses’ total IIS scores and gender, job satisfaction, and following innovations. Today, there are different views on the effect of gender on innovativeness. In this context, studies revealing that males are more innovative similar with the findings of this study [12]. While males are expected to exhibit more cyberloafing behaviors due to the higher rate of internet use (89.1%), in this study, no significant difference was found in the level of cyberloafing according to the gender variable [23]. However, in some studies, it was found that men [44, 45] and in some studies, women's cyberloafing levels are higher [43]. In line with the findings obtained as a result of a detailed literature review, it is seen that cyberloafing behaviors of health workers, especially nurses, are compared according to some characteristics, but different results are obtained. It is thought that the different sample groups are effective in the emergence of this result.

It was determined that those who were very satisfied with their profession and follow innovations partially belonged to the category of “inquirers”, that they were more innovative than the others. Similarly, Sonmez and Yildirim (2014) reported that innovative behaviors were affected by individual characteristics and motivation [39].

It was revealed that the nurses’ age, working year, and period of daily internet use had an impact on their cyberloafing behaviors. The nurses aged 36 and over who had worked for over 11 years displayed low levels of cyberloafing behaviors, and the period of daily internet use increased, and cyberloafing behaviors increased. Tatli et al. (2018) revealed a negative relationship between nurses’ age and working year and their levels of utilizing information technologies [46]. The study of Opucu and Yildiz (2014) which determined that cyberloafing behaviors were affected by age and daily period of internet use, also supports the findings of this study. In the study of Arslan and Demir (2016), it was reported that the cyberloafing levels of single, childless and low-income nurses are higher. Mc Bride et al. (2015) compared to Arslan and Demir (2016) under the age of 30 [13], cyberloafing behavior is significantly more common in nurses under the age of 20. Amarat et al. (2017), on the other hand, it was reported that the Y generation engaged in more cyberloafing behavior than the X generation.

As the period spent in front of a computer increases, cyberloafing during this period may be regarded as a right [37]. These technologies, which are a part of work, may arouse the feeling in the user that performing loafing activities is permissible provided that his/her tasks are completed on time [37, 47]. Similarly, in Bacaksiz et al. (2018) study, it was concluded that participants with more working experience had lower mean scores of cyberloafing [40]. In a study conducted with nursing students, it was determined that with the increase in the use of smartphones in the clinics, the cyberloafing behaviors of the students increased, but their clinical decision-making skills decreased [48]. In the study of Syed et al. (2020), it was stated that cyberloafing has both positive and negative effects on job performance [49]. It is suggested that cyberloafing is not to be completely prevented, but to be implemented within acceptable limits for employees and employers [49]. The research result of Rahman et al. (2020) also supports this result [50]Consistent with previous studies, the findings of this study suggest that less working experience may have caused the lack of sense of corporate belonging and professional responsibility and the use of cyberloafing behaviors may have been used as an individual coping method during this period. There are also studies reporting that the cyberloafing behavior levels of employees in private health institutions are low [1]. In addition, cyberloafing levels of administrators and administrative staff were found to be higher than health workers [1, 21].

In the analysis based on the view that innovative individuals can perform cyberloafing as a form of learning, it has been concluded that individual cyberloafing behavior is not associated with innovativeness. Besides the studies reporting negative effects of cyberloafing [26, 27, 41], there have been also reported positive effects of it, like innovativeness [7, 51]In the study of Zhong et al. (2022), which examined the effect of cyberloafing on the innovative performance of employees during the COVID-19 epidemic in the context of the theory of conservation of resources (COR), it was stated that working from home increased the cyberloafing behavior within the scope of pandemic measures [52]. In particular, surfing websites to obtain information about the COVID-19 disease has become a common form of cyberloafing. Virtual loafing exhibited during the pandemic period; It has been stated that it has a positive effect on gratitude, the sense of meaningfulness of work, and innovative performance [52]. According to the results of Tsai's (2023) study in which he examined the cyberloafing behaviors of employees in the context of the conservation of resources (COR) theory; It has been argued that cyberloafing increases productivity by drawing the attention of individuals in another direction and it has a positive effect on innovation [53]. In the study of Karabulut and Akar (2020) with pre-service teachers, it was determined that there was a weak positive correlation between the innovative characteristics and cyberloafing behaviors of the participants [53, 54].

There are studies in the literature stating that cyberloafing can yield positive results such as productivity, stress reduction, and job satisfaction [55, 56]. Therefore, evaluating cyberloafing as a negative behavior would not be realistic.

Organisations that wish to adapt to the evolving technological landscape and effectively regulate the phenomenon of cyberloafing must first comprehend the concept of cyberloafing, which is characterised by a complex structure. In order to mitigate the negative consequences of cyberloafing while capitalising on its potential benefits, it is essential to develop strategies that address this multifaceted issue [57]. It is important to develop clear guidelines, work ethics and policies to limit employees' cyberloafing behaviour. Managers should determine what levels and types of cyberloafing will be tolerated and provide training programmes and counselling to employees accordingly [5, 24, 28].

In order to limit cyberloafing behaviour, institutions can expand their interventions on monitoring and blocking modules for internet use and approved websites. In addition, implementing a punishment and reward strategy and establishing a fair complaint and appeal process for sanctions can support the development of self-control and self-regulation behaviours in employees [24, 28].

Creating a daily goal for employees and setting a deadline for the completion of the task are among the measures that can be taken to reduce cyberloafing behaviour. In addition, directing the computer screen to the corridors instead of the walls to increase visibility will limit cyberloafing behavior [28, 57].

Considering the influence of individual characteristics on cyberloafing behaviour, it should be noted that some employees may be psychologically more prone to cyberloafing. Employers need to assess potential candidates based on their broad psychological profile, and to examine personality traits and pre-existing problems related to internet addiction in depth. It is recommended that future research adopt an interdisciplinary approach to assess the psychology, communication norms and media preferences underlying cyberloafing behaviour [28, 58].

With the results of the study, it is thought that increasing the awareness level of individuals towards cyberloafing behavior will reduce the negative effects of cyberloafing.

## Conclusions

The nurses’ innovativeness characteristics were at a low level, and they did not frequently perform cyberloafing behaviors. Moreover, as there was no correlation between cyberloafing and individual innovativeness, cyberloafing was not an indicator of innovativeness.

The cyberloafing and innovativeness levels of nurses were found to be low. It has been determined that innovativeness features are affected by ‘gender’, ‘job satisfaction’ and ‘following innovations’, and cyberloafing behaviors are affected by ‘age’, ‘working years’ and ‘daily internet use’. In addition, since there is no relationship between cyberloafing and individual innovativeness levels, cyberloafing is not an indicator of innovativeness.

### Implications for nursing practice

Institutional regulations and initiatives can be recommended for fostering more conscious cyberloafing that will give nurses the opportunity for individual and professional self-improvement in the context of innovativeness. Cyberloafing can be a tool for individuals, especially those with high levels of personal innovativeness, to follow and create innovations. The positive and negative effects of cyberloafing should be examined in more detail, and individuals should be supported in the development of their innovativeness characteristics by setting the limits of acceptable cyberloafing behavior. Moreover, future research studies can contribute to the literature by evaluating the cyberloafing behaviors of innovative individuals.

### Limitations

This study’s results are limited to the self-assessed views of the nurses working at the hospital in which the research was conducted. Findings from the study should not be generalized to the whole population due to limited sample representation. In addition, since the study is based on self-report, there may be a case of misrepresentation due to forgetting. Finally, the level of cyberloafing and innovativeness was evaluated from the perspective of the nurse, not from the perspective of managers, colleagues, or employers. This is among the limitations of the study as it may cause bias. Nevertheless, this study is one of the few in which the relationship between cyberloafing and innovativeness has been investigated.

## Data Availability

The data used to support the findings of this study are available from the corresponding author upon reasonable request.

## Abbreviations

IIS: Individual Innovativeness Scale
CLS: Cyberloafing Scale
